# A feasibility trial of a psychological intervention for addressing alcohol problems during pregnancy: a study protocol

**DOI:** 10.1186/s40814-026-01932-5

**Published:** 2026-10-09

**Authors:** Petal Petersen Williams, Isabelle L. Lange, Jodilee Erasmus, Lesley-Ann Erasmus-Claassen, Maham Saleem, Lisa Sophie Reigl, Meike Wolf, Stefan Schaub, Christoph Buck, Abhijit Nadkarni, Daniela C. Fuhr

**Affiliations:** 1 Public Health Research Department, Faculty of Medicine and Health Sciences, University of East Anglia, Norwich Research Park, Norwich, NR4 7TJ UK; 2 Mental Health, Alcohol, Substance Use and Tobacco Research Unit, South African Medical Research Council, PO Box 19070, Francie Van Zijl Drive, Parowvallei, Cape Town, Tygerberg 7505 South Africa; 3 Institute for Life Course Health Research, Department of Global Health, Stellenbosch University, Francie Van Zijl Drive, Cape Town, Tygerberg 7505 South Africa; 4 Department of Neurology, TUM University Hospital and Center for Global Health, School of Medicine and Health, Technical University of Munich, Ismaninger Straße 22, Munich, 81675 Germany; 5 Department of Infectious Disease Epidemiology and International Health, London School of Hygiene & Tropical Medicine, Keppel Street, London, WC1E 7HT UK; 6 Leibniz Institute of Prevention Research and Epidemiology - BIPS, Achterstraße 30, Bremen, 28359 Germany; 7 Unit Research Coordination, Cooperation, Knowledge Transfer, Federal Institute of Public Health, BIÖG, Maarweg 149–161, Cologne, 50825 Germany; 8 Centre for Global Mental Health, Department of Population Health, London School of Hygiene and Tropical Medicine, Keppel Street, London, WC1E 7HT UK; 9 Addictions and Related Research Group, Sangath, H No 451 (168), Bhatkar Waddo, Socorro, Bardez, Porvorim, Goa 403501 India; 10 Health Sciences, University of Bremen, Mary-Somerville-Straße 3, Bremen, 28359 Germany; 11 Department of Health Services Research and Policy, London School of Hygiene and Tropical Medicine, Keppel Street, London, WC1E 7HT UK

**Keywords:** Pregnant women, Alcohol, Counselling, Community health workers (CHWs), South Africa

## Abstract

**Background:**

South Africa has the highest global prevalence of foetal alcohol spectrum disorders (FASD). Despite guidelines for FASD prevention, there are no community-based psychological interventions to reduce alcohol use during pregnancy. Through formative research, a culturally adapted intervention, the Mommy and Baby Wellness Programme, was developed to address the needs of pregnant women in the setting. The aim of the feasibility trial is to refine the trial methods before conducting a full-scale trial, and explore the feasibility of programme delivery, the acceptability of session content, and participants’ perceptions of its effectiveness.

**Methods and analysis:**

A parallel-arm, single-blind, individually randomised feasibility trial will be conducted. Forty pregnant women (≥ 18 years) screening positive for hazardous alcohol use (AUDIT score ≥ 8) and confirming past-month alcohol consumption will be randomised 1:1 to receive either the Mommy and Baby Wellness Programme or enhanced usual care. Women will be assessed at baseline and again three months after randomisation. The study uses an embedded mixed-methods design, in which a parallel quantitative feasibility trial is complemented by qualitative interviews with participants and community health workers. Quantitative indicators of trial feasibility, acceptability and implementation (e.g. recruitment, retention, adherence and completeness of outcome data) will be summarised descriptively, and qualitative data will be used in an explanatory role to help interpret these findings and refine trial procedures and intervention delivery. Ethical approval has been obtained from the South African Medical Research Council (SAMRC) and the Technical University of Munich Health Research Ethics Committees. All participants will provide informed consent. Study data will be securely managed using *R*esearch *E*lectronic *D*ata *Cap*ture software hosted on SAMRC servers.

**Discussion:**

The pilot trial of the Mommy and Baby Wellness Programme provides initial evidence on the feasibility and acceptability of a community health worker–delivered psychological intervention to reduce alcohol use among pregnant women in South Africa. Findings will inform the design of a future definitive trial and guide adaptation of recruitment, retention, and counselling strategies for a highly stigmatised population. Insights from the process evaluation will refine approaches to participant engagement and fidelity monitoring, supporting culturally responsive and sustainable implementation. If feasible, the intervention could contribute to improved maternal and child health outcomes and reducing alcohol-related harm in resource-limited settings.

**Trial registration:**

ClinicalTrials.gov ID NCT06853873. Retrospectively registered: 2025-02-25 https://www.clinicaltrials.gov/study/NCT06853873

**Supplementary Information:**

The online version contains supplementary material available at https://doi.org/10.1186/s40814-026-01932-5.

## Background

The harmful use of alcohol is a leading risk factor for population ill-health worldwide [1], with low- and middle-income countries (LMICs) disproportionately affected [2]. Substance use is recognised as a key development issue and ensuring access to prevention and treatment of alcohol and other substance use disorders is listed as one of the Sustainable Development Goals [3]. Problem alcohol use has negative social and economic impacts for the individual (e.g. loss of work, social isolation, reduced productivity and income) and is therefore strongly linked with poverty [3]. South Africa (SA) is estimated to have the highest prevalence of foetal alcohol spectrum disorders (FASD) in the world with 111 cases per 1000 population [4]. FASD is an umbrella term which describes several conditions associated with foetal alcohol exposure (foetal alcohol effects, partial foetal alcohol syndrome, alcohol-related birth defects and alcohol-related neurodevelopmental disorder [5]).

Despite some efforts to address the high prevalence of FASD and the availability of guidelines for the prevention and management of FASD [6], no national policies or scalable community-level interventions are available in SA. Services to detect alcohol use during pregnancy and to prevent alcohol-exposed pregnancies are largely absent outside of antenatal services offered through primary healthcare (PHC) clinics [7].

Counselling for Alcohol Problems (CAP) is an evidence-based, manualised, brief psychological counselling therapy for alcohol misuse, based on principles of motivational interviewing and cognitive-behavioural strategies such as problem-solving [8]. CAP is currently the only available evidence-based alcohol-focused brief therapy designed to be delivered in community settings by lay counsellors in a low- and middle-income country (LMIC) [9] and was highly effective in reducing alcohol use at 3-months and 12-months among men in India [8, 10].

Building on extensive formative research with pregnant women in South Africa [9, 11], we adapted CAP for the antenatal context. In line with provincial health department community health worker (CHW) programmes [12, 13] to expand community-based maternal and child health services, our aim was to develop an intervention that is both theoretically coherent and pragmatically aligned with the existing community health worker platform. Community health worker delivery was therefore chosen to integrate with current provincial service models rather than to introduce a parallel, stand-alone service. The formative work comprised qualitative interviews and focus group discussions with pregnant women, partners and CHWs, as well as co-design workshops with local stakeholders, to adapt the CAP intervention content, examples and delivery format to the local antenatal and community context. The study is situated in a context characterised by high levels of poverty, structural inequality, limited access to health and social care, and stigma related to alcohol use during pregnancy. These conditions create important health inequities in who is reached by existing services and who can benefit from new interventions. In designing and adapting the intervention, particular attention was given to accessibility for socially and economically disadvantaged women, including community-based, healthcare worker-delivered counselling and flexible scheduling of sessions. The overall aim of study is to assess the feasibility of trial processes and evaluate early implementation outcomes in preparation for a future definitive randomised controlled trial (RCT). Specific objectives are:

1. Trial procedures—To pilot, and if needed, refine procedures for screening, recruitment, randomisation, retention, and follow-up assessments, and to determine the feasibility of data collection methods for use in a definitive RCT.
2. Implementation outcomes—To assess the feasibility of delivery, acceptability of session content, and safety of delivering the intervention in combination with enhanced usual care (EUC).
3. Preliminary signals of effectiveness—To explore perceived effectiveness of the intervention on participants’ alcohol use and mental health outcomes.

## Methods and analyses

The protocol is reported in accordance with recent guidelines for pilot and feasibility trial protocols [14]. This includes the SPIRIT (Standard Protocol Items: Recommendations for Interventional Trials) checklist [15] and the CONSORT 2010 statement: extension to randomised pilot and feasibility trials [16]. See Additional file 1 for SPIRIT checklist.

We begin by outlining the design and methods of the feasibility trial, followed by a description of the approaches used to assess the feasibility trial objectives and finally, a summary of the planned analyses.

### Trial design and procedures

**Fig. 1:**
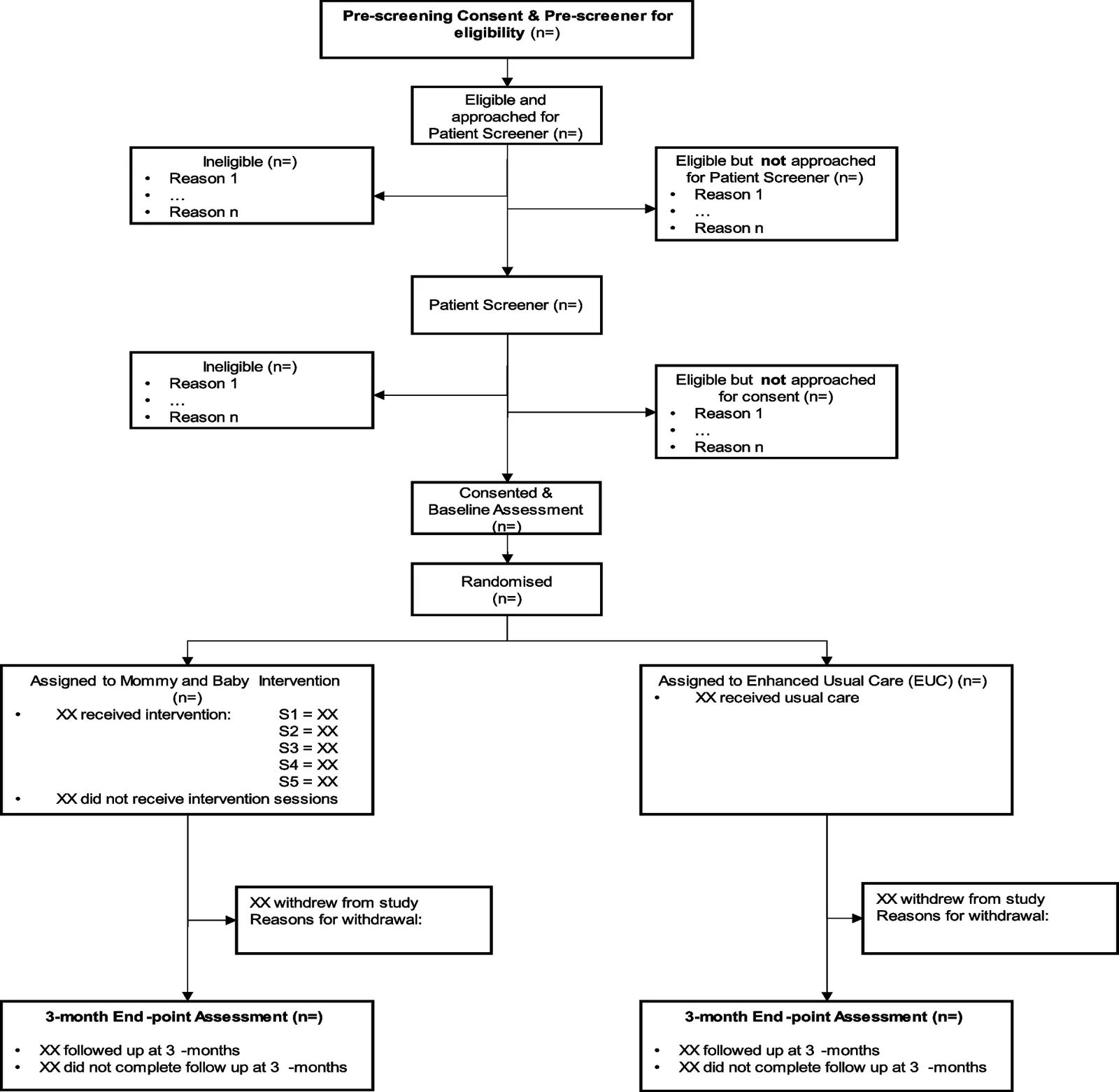
The Consolidated Standards of Reporting Trials (CONSORT) diagram

A parallel-arm, single-blind, individual-randomised feasibility trial will be conducted, using an embedded mixed-methods design with qualitative interviews nested within the trial. The qualitative component is primarily explanatory, designed to explore participants’ and providers’ experiences of trial procedures and intervention delivery, and to aid interpretation of quantitative feasibility indicators during the overall synthesis of findings. The Consolidated Standards of Reporting Trials (CONSORT) diagram is presented in Fig. 1.

### Study setting

The study will take place in low-income communities in the Cape Metropole of Western Cape, South Africa, The prevalence of FASD is particularly high (20–28%) in the wine-growing region of the Western Cape (WC) of SA [17–19]. Drinking alcohol among pregnant women is common there, particularly in the impoverished townships of the WC, where prevalence of alcohol consumption (including heavy episodic drinking) has ranged from 20% to 61% across various studies covering a range of measures of frequency and quantity [20–22]. Health and social support services for individuals with alcohol-related problems are limited but include primary healthcare clinics, community-based NGOs, and government-funded substance abuse treatment centres located mainly in urban areas and are not always consistently available or tailored for pregnant women.

### Participant recruitment and study procedures

#### Identification and screening for eligibility

To be eligible for inclusion in this study, women must be aged > 18 years, < 28 gestational weeks pregnant, score 8–20 on the Alcohol Use Disorder Identification Test (AUDIT) (i.e. hazardous/harmful drinking) and self-report past month alcohol use. We will exclude those currently receiving mental health services, or report that they have any serious medical problems or comorbid conditions. Women currently receiving mental health services are excluded from this initial feasibility trial to avoid conflicts or duplication between existing care and the study intervention, and because they may have more complex clinical needs than can be safely addressed within a brief, community health worker (CHW)-delivered programme. This decision reduces heterogeneity in the sample to allow clearer assessment of trial procedures and early implementation outcomes. We recognise that this may limit external validity and plan to reconsider inclusion criteria in light of feasibility and safety findings when designing a future definitive trial. Pregnant women will be identified through community-based outreach strategies, with support from community leaders and CHWs. Outreach activities will include brief information sessions at existing community gatherings (e.g. women’s groups, faith-based meetings and maternal and child health events), distribution of flyers and posters in community venues (e.g. clinics, community centres and shops), and door-to-door or home-visit contacts by CHWs within their catchment areas. CHWs and community leaders will introduce the study using a standardised script that emphasises voluntary participation, confidentiality and the non-judgemental nature of the counselling, in order to reduce stigma related to alcohol use in pregnancy. Recruitment activities will be reviewed regularly by the study team to monitor the number of women approached, screened and enrolled, and to adapt outreach strategies if recruitment falls below feasibility targets. Research staff will approach potential participants, explain the study, and complete a pre-screening consent form before administering a brief pre-screener to assess basic eligibility outlined above. Women who are potentially eligible will be invited to be transported to the South African Medical Research Council (SAMRC) clinical research site in Delft for a confirmatory urine pregnancy test and formal screening against study eligibility criteria. If the test is negative, i.e. participants are found not to be pregnant, participants will be returned to the recruitment site and provided with a ZAR100 voucher to thank them for their time. Snowball sampling will also be used, with initial participants referring to peers who may be eligible. Snowball sampling is used as a pragmatic strategy to reach women who may not be easily identified through routine community platforms because of stigma surrounding alcohol use in pregnancy. We recognise that this approach may introduce self-selection bias and limit representativeness of the sample. However, in this feasibility trial, the primary objective is to assess the practicality and acceptability of recruitment procedures and trial processes in a highly stigmatised population, rather than to obtain population-representative estimates.

#### Informed consent and baseline data collection

Eligible participants who provide written informed consent will be recruited in the study and complete a baseline questionnaire assessing demographic and socioeconomic characteristics, alcohol use history, physical and mental health indicators, and relevant psychosocial factors. Baseline assessment also includes the collection of dried blood spot (DBS) samples for phosphatidylethanol (PeTH) testing. The baseline assessment will take place in a private room at the SAMRC clinical research site. Data will be collected using secure, password-protected electronic tablets.

After completing the baseline assessment, participants will be randomised to either the intervention (Mommy and Baby Wellness Programme) or the control (enhanced usual care) arm. Where possible, participants randomised to the intervention arm will receive Session 1 of the programme on the same day. The primary endpoint will be at three months post-randomisation. Therefore, for this feasibility trial, quantitative outcome assessments are limited to baseline and three-month follow-up. This schedule was chosen to balance participant burden and resource constraints in a highly stigmatised, hard-to-reach population, while still providing an early indication of potential changes in alcohol use and mental health. This feasibility trial is not designed to assess long-term sustainability of effects. Findings on recruitment, retention and data completeness at these time points will inform whether additional measurement occasions are feasible and warranted in a future definitive trial and extend follow-up into later pregnancy and/or the postpartum period.

### Randomisation and blinding

Forty pregnant women will be randomised using a 1:1 allocation ratio via a computer-generated randomisation sequence managed by an independent statistician. The allocation sequence will be concealed using sequentially numbered opaque sealed envelopes. This study will employ single-blind masking. Due to the nature of the intervention, participants and counsellors will not be blinded to treatment. Data collectors and assessors conducting follow-up evaluations will be blinded to treatment allocation. This ensures objective data collection and reduces potential observer bias in outcome assessments. To maintain blinding, assessors will not be involved in intervention delivery and will not have access to participant allocation records. Because participants know their allocation and may inadvertently reveal it during follow-up contacts, there is a risk that assessor blinding could be compromised. To minimise this, assessors will be trained to avoid probing about counselling experiences and to record any instances where allocation is disclosed, so that the extent of unblinding can be considered when interpreting feasibility and outcome data.

### Intervention

#### Control arm: enhanced usual care

Participants randomised to the control arm will receive enhanced usual care (EUC). EUC is treatment as usual enhanced by an information sheet describing what pregnant women can do to lower their drinking and where to get community support and referral to antenatal care. EUC was selected as the comparator because it reflects the locally available standard of care for pregnant women who use alcohol and therefore provides an ethically appropriate minimum level of information and referral support to all participants. In the context of a feasibility trial, EUC allows us to test recruitment, randomisation, blinding and follow-up procedures while maintaining a clear distinction between the structured psychological intervention and usual care. To standardise EUC across participants, counsellors will use a brief, structured information sheet and a consistent set of referral options to antenatal and community services; any deviations from this protocol will be recorded in field notes.

#### Intervention arm: Mommy and Baby intervention

Participants assigned to the intervention group will receive EUC and up to five weekly sessions of the Mommy and Baby Wellness Programme delivered by a trained CHW. The Mommy and Baby Wellness intervention was adapted from the original CAP intervention, which targets men with problem alcohol use in India [8], to address hazardous/harmful alcohol use among pregnant women in South Africa. Core therapeutic elements of CAP were retained, including the collaborative, motivational interviewing–informed counselling style, a structured sequence of sessions, and the use of cognitive-behavioural strategies such as problem-solving, drink tracking, goal-setting and relapse prevention exercises. For the antenatal context, session content was modified to place greater emphasis on pregnancy-specific psychoeducation about alcohol use and foetal and maternal health, exploration of ambivalence about drinking in relation to the unborn baby, and planning for safer alcohol use or abstinence within women’s family and relationship contexts. Some CAP content was streamlined to prioritise pregnancy-related concerns and to fit within up to five weekly sessions. The adaptation process was grounded in community-based participatory research and comprised multiple stages designed to ensure contextual and cultural relevance. Adaptation was informed by prior formative research with pregnant women and healthcare providers in the study communities, which explored barriers and facilitators to disclosing alcohol use and engaging with counselling, as well as preferences for delivery format and content. Iterative feedback on draft session outlines and client materials was obtained through workshops with pregnant women, community health workers and local stakeholders, and revisions were made to language, examples and exercises to enhance cultural resonance and acceptability. This community-based participatory approach ensured that the core mechanisms of CAP were preserved while tailoring the intervention to the specific needs, values and constraints of pregnant women in South Africa. Sessions of the Mommy and Baby Wellness Programme are organised into three phases: (i) awareness and motivation to change, (ii) behaviour change planning and support, and (iii) relapse prevention and consolidation. The Mommy and Baby client booklet complements each session and provides culturally tailored tools, including drink tracking, goal-setting templates, psychoeducation on maternal and foetal health, strategies for coping with cravings and difficult emotions, as well as relapse prevention exercises. The intervention is informed by a theory of change in which structured counselling aims to increase women’s motivation and confidence to reduce or stop alcohol use during pregnancy, build practical skills for managing cravings and stress, and strengthen social support for behaviour change. In Phase 1, motivational interviewing techniques are used to explore ambivalence, elicit change talk and enhance perceived importance and self-efficacy for changing alcohol use. In Phase 2, cognitive-behavioural strategies such as goal-setting, problem-solving and coping skills are used to support implementation of change plans in the context of women’s everyday stressors and relationships. In Phase 3, relapse prevention and consolidation exercises focus on anticipating high-risk situations, rehearsing coping responses and reinforcing non-drinking goals to support maintenance of change beyond the counselling period. The Mommy and Baby Wellness Programme therefore combines core elements of motivational interviewing (empathic, collaborative counselling to resolve ambivalence) with cognitive-behavioural counselling techniques (structured goal-setting, problem-solving and coping skills training) that were central to the original CAP intervention and adapted here for pregnant women in South Africa.

The intervention will be delivered in person by trained CHWs at the SAMRC’s community-based research site. Service planners in the WC recognise that, to reach at-risk pregnant women for psychological counselling, they need to move beyond only offering services in PHC clinics to include services that reach women in their communities. Given shortages of healthcare professionals, these community-based antenatal services have been task-shared from nurses and midwives to trained CHWs. While CHWs undergo training in maternal and child healthcare, this training does not address how to recognise or address alcohol use among pregnant women, partly because there is a dearth of evidence-based programmes to address alcohol use during pregnancy that are acceptable to pregnant women, feasible for CHW delivery and suitable for the local context.

For the study, selected CHWs will undergo a comprehensive training about the intervention’s content and delivery model. The training will include core counselling competencies such as active listening, empathy, motivational interviewing techniques, as well as strategies to build client engagement. CHWs will also be trained in the specific content of the Mommy and Baby manual and the use of the Mommy and Baby client booklet. Intervention fidelity will be assessed using a structured checklist completed by an experienced clinical supervisor or qualified counsellor while reviewing a sample of audio-recorded sessions (with participant consent). The checklist will capture adherence to core session components, use of key counselling techniques (e.g. motivational interviewing strategies), and overall quality of delivery. Supervisors will provide written and verbal feedback to CHWs in bi-weekly supervision meetings, and brief competency assessments will be repeated periodically to identify training needs and maintain consistent delivery across counsellors.

### Proposed trial outcome measures

**Fig. 2:**
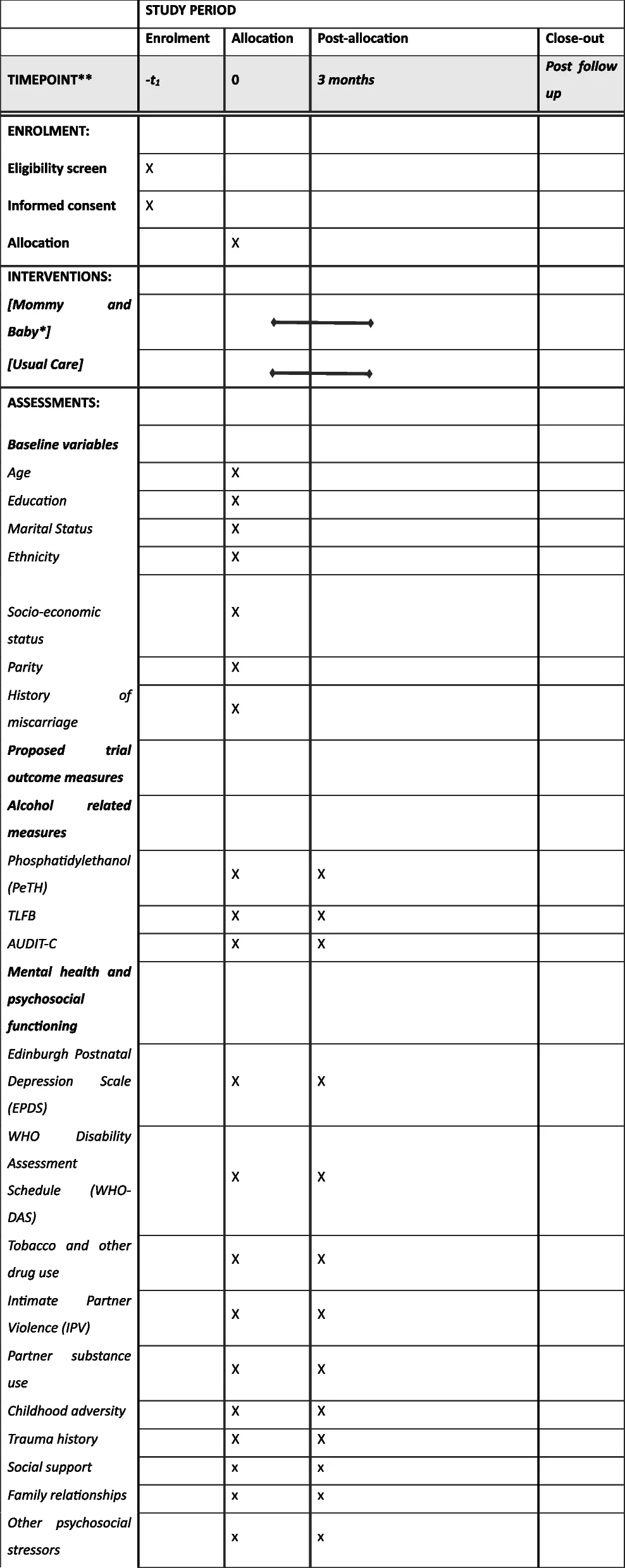
Standard Protocol Items: Recommendations for Interventional Trials (SPIRIT) figure—schedule of enrolment, intervention and data collection

The schedule of data collection is shown below in Fig. 2 SPIRIT figure—schedule of enrolment, intervention and data collection. The outcome measures were selected to align with the intervention’s theory of change. Alcohol-related measures capture the primary behavioural target of the intervention, namely reductions in frequency and quantity of alcohol use during pregnancy. Mental health and psychosocial functioning reflect anticipated improvements in mood and daily functioning arising from enhanced coping skills, problem-solving and reductions in alcohol-related harm. Additional psychosocial variables, including tobacco and other drug use, intimate partner violence, partner substance use, social support, family relationships and other stressors, provide contextual information on the relational and environmental factors that are addressed in counselling and may influence women’s ability to change their drinking.

Primary outcome measures: Self-reported alcohol use measured using the Alcohol Timeline Follow-Back (TLFB) method, which provides retrospective estimates of daily alcohol consumption [23].

Secondary outcome measures: Phosphatidylethanol (PeTH), a direct biological marker of alcohol consumption, will be assessed using a finger-prick dried blood spot test (DBS), as a clinical outcome. This biomarker offers high sensitivity and specificity for recent alcohol use and can reliably distinguish between heavy, moderate, and no alcohol use within the prior 2–3 weeks [24]. PeTH will be used to objectively validate self-reported alcohol consumption and to monitor reductions in drinking over time. We will also use the AUDIT-C [25], a brief version of the Alcohol Use Disorder Identification Test (AUDIT), to assess severity of drinking.

Mental health and psychosocial functioning: Depression symptoms will be assessed using the Edinburgh Postnatal Depression Scale (EPDS) [26], a validated measure for detecting perinatal depression. Psychosocial functioning will be measured with the WHO Disability Assessment Schedule (WHO-DAS) [27], which assesses health and disability across six domains of functioning. Additional variables will include tobacco and other drug use measured using the Drug Use Disorders Identification Test (DUDIT) [28], intimate partner violence (IPV), partner substance use, childhood adversity, trauma history, social support, family relationships, and other psychosocial stressors using a bespoke questionnaire.

Demographic and contextual variables: At baseline, participants will provide demographic and obstetric information, including age, education, socioeconomic status, parity, history of miscarriage, and perceived usefulness of counselling.

In this feasibility trial, these instruments are used primarily to assess the acceptability and practicality of collecting outcome data in this population, and to provide preliminary information on whether the selected measures are responsive to the mechanisms targeted by the intervention. Reductions in alcohol use will be quantified as changes in TLFB-derived indicators (e.g. mean number of drinks per week, number of heavy drinking days) and in PeTH categories between baseline and three-month follow-up, providing both self-report and biological evidence of change in drinking patterns as preliminary signals of effectiveness on alcohol, rather than against a prespecified minimum clinically important difference. Findings from this study will inform refinement of the outcome set and the choice of primary and secondary endpoints for a future definitive randomised controlled trial.

### Sample size considerations

The planned sample size of 40 participants (20 per arm) is appropriate for a feasibility trial focused on estimating key process parameters rather than detecting intervention effectiveness. In line with recommendations for pilot and feasibility studies, this group size allows reasonably precise estimation of recruitment, retention and adherence rates, and provides initial estimates of outcome variability to inform the sample size calculation for a future definitive trial. The choice of 40 participants also reflects pragmatic considerations, including the expected pool of eligible pregnant women with hazardous alcohol use in the study communities, anticipated stigma-related barriers to disclosure and participation, and available resources for trial delivery.

### Data management

All study data will be collected and managed using REDCap, a secure, web-based system hosted by the SAMRC. Data will be stored in line with institutional policies to ensure confidentiality and security. Each participant will be assigned a unique study ID, and no personal identifiers will be stored with study data. Data quality will be monitored through regular checks, including range and consistency checks and periodic review of recruitment numbers and completeness of key outcome variables. Any data queries identified will be resolved by the study team through verification against source documents. Contact details and locator information will be held separately in restricted, password-protected files. Qualitative data will be transcribed with identifying information removed. Paper-based documents (e.g. consent forms, test results) will be stored securely at the research site. Access to pseudonymised study data will be limited to authorised personnel, and all outputs or publications will use anonymised or aggregated data only. De-identified trial data and accompanying documentation may be made available from the corresponding author upon reasonable request, subject to ethical and institutional approvals. Access will be limited to authorised researchers under appropriate data-sharing agreements.

### Trial management

The project will be governed by a Project Management Group (PMG). The PMG will be chaired jointly by the two principal investigators (PIs) whose membership will include co-investigators (Co-Is) and all research staff. The PMG will advise on the oversight and coordination of programme management, communication and dissemination, publications and data sharing, and integration and optimal utilisation of programme resources. To further protect against any breaches of confidentiality, all project staff proposed to conduct data collection have received or will receive training on Good Clinical Practice (GCP) and Quality Assurance.

Given the nature and scope of this feasibility trial, a formal Data and Safety Monitoring Board (DSMB) will not be established. Safety oversight will instead be provided through the following mechanisms: 1) PI and study team monitoring participants for adverse events throughout the study; and 2) Institutional Review Board (IRB) and Human Research Ethics Committee (HREC) will review and approve the protocol, and all adverse events (AEs) will be reported according to institutional and regulatory requirements. Due to the vulnerability of pregnant women who use alcohol, clear criteria will guide any modification or discontinuation of intervention delivery. If a participant experiences serious distress, discloses acute risk (e.g. severe intimate partner violence, suicidality) or an adverse event judged to be related to the intervention, CHWs will pause the session, consult the clinical supervisor, and prioritise referral to appropriate health or social services. The Project Management Group will review such cases and may recommend adaptations to scheduling, content emphasis, or additional safety checks, documenting all changes. These procedures ensure that intervention continuity is balanced with participant safety.

Alcohol use during pregnancy is a very sensitive topic and many participants will be socially vulnerable. Therefore, potential risks include feelings of guilt or shame, fear of judgement, and, in some cases, heightened tension or conflict within families or relationships. To minimise these risks, recruitment and counselling contacts will be conducted in private settings where possible; staff will use non-judgemental, supportive language that emphasises health promotion rather than blame; and participation will be framed as engagement in a general maternal and child health programme rather than as ‘treatment for alcohol problems’. Field staff and counsellors will receive training on recognising distress, responding to sensitive disclosures, and avoiding actions that could inadvertently increase stigma or social risk.

In community settings where privacy may be limited, staff will take steps to protect confidentiality, such as arranging individual conversations away from bystanders and avoiding discussion of alcohol use within earshot of others. Sensitive information about alcohol use, violence or other psychosocial stressors will not be shared with family members or community members without the participant’s explicit consent, except where mandatory reporting requirements apply. Clear procedures will guide staff responses to disclosures of intimate partner violence, severe emotional distress or other social risks, including provision of immediate support, safety planning where appropriate, and referral to available health and social services. These ethical safeguards are integrated with our adverse-event monitoring and referral procedures, ensuring that any social or psychological harms identified during the study are responded to promptly and inform adjustments to recruitment and counselling strategies.

### Adverse events

For the proposed study, AEs include any of the following outcomes for the participant: (1) death, (2) acute life-threatening incidents, (3) hospitalisation or the prolongation of a hospitalisation, and/or (4) persistent or significant disability or incapacity. They will be reported regardless of whether they are considered to be study related. If participants experience any adverse reactions to any aspects of the study, the project staff will contact the PI immediately for additional support and will notify the other senior staff via an incident report. Based on advice from senior project staff, the field staff will make recommendations for appropriate referrals to counselling and/or other health services. The incident reports will be reviewed weekly by the PIs. All AEs will be immediately reported to the SAMRC’s HREC, following standard reporting procedures. Adverse events will be summarised descriptively by type, severity and relationship to study procedures or intervention delivery. Patterns of AEs will be reviewed by the project management group and ethics committees to inform safety monitoring plans for any future definitive trial.

## Methods to assess feasability objectives

### Primary feasibility outcomes

In line with recommendations for the reporting of protocols of pilot and feasibility trials [14], we will collect both quantitative and qualitative data to determine the acceptability and feasibility of intervention delivery and trial procedures. Within the embedded mixed-methods design, quantitative data on feasibility progression criteria will be analysed first using descriptive statistics. Qualitative interview data from participants and community health workers will then be analysed thematically and used to explain, contextualise, or challenge the quantitative feasibility indicators. Integration will occur at the interpretation and reporting stages, where quantitative and qualitative findings will be triangulated to inform decisions about adapting trial procedures for a future definitive trial. Specifically, we will assess trial procedures including recruitment (including numbers screened, enrolled, and reasons for refusal), randomisation, retention and follow-up rates, and completeness of outcome data. Intervention delivery and implementation outcomes will include acceptability of session content to both participants and providers, feasibility of delivery, alongside monitoring for adverse events. Qualitative feedback will provide insights into participants’ and providers’ perceptions of the intervention’s usefulness and potential impact on alcohol use and mental health. Objectives of our feasibility trial, including their assessment criteria, data collection methods, time of assessment, and target (progression criteria for a full trial), are outlined in Table 1 and are adapted from Fuhr et al. [29]. Feasibility criteria for progression to a full trial were informed by Mellor et al. [30]. To aid interpretation, we organise feasibility indicators into three overarching domains: (1) trial feasibility, referring to the extent to which key trial processes (screening, recruitment, randomisation, retention and data collection) can be successfully implemented in this setting; (2) intervention acceptability, referring to participants’ and providers’ perceptions that the intervention content, format and burden are agreeable and satisfactory; and (3) implementation feasibility, referring to the practicality of delivering the intervention as intended by community health workers within existing service structures. This organisation is informed by commonly used feasibility and implementation frameworks, in which feasibility focuses on whether a study or intervention can be done, acceptability on how it is experienced, and implementation on the fidelity and practicality of delivery in real-world conditions. Within each domain, quantitative indicators (e.g. recruitment rates, retention, adherence, fidelity scores and data completeness) will be considered alongside qualitative accounts from participants and providers to build an integrated picture of feasibility. Patterns across domains will be used to identify aspects of trial procedures and intervention delivery that require modification before progressing to a definitive trial.

**Table 1:** Mommy and Baby Wellness Programme feasibility study progression criteria (adapted from Fuhr et al. [29])

Objectives of feasibility trial	Assessment criteria	Method	Time of assessment	Target
1. **Piloting trial procedures**				
**Recruitment:** To investigate recruitment and expected recruitment timeline to reach target sample size	- Number of participants approached - Number assessed for eligibility - Number eligible - Recruitment rate per month - Number consented or enrolled - Number of dropouts before randomisation	- Raw count (number or %), and calculation of recruitment rate and duration (participants enrolled per month)	- Interim assessment (continuous monitoring throughout the recruitment phase)	- ≥ 60% of eligible women enrolled (target N = 40)
**Randomisation:** To investigate if randomisation was successful	Randomisation process	Checking the correct use of random number generator, and allocation concealment	At the start of randomisation	No incorrect use of random numbers
**Retention:** To investigate how well participants stayed in the trial through follow-up	- Overall retention rate - Overall drop-outs by sub-group (e.g. age, ethnicity, education, marital status, employment) - Timing of drop out in intervention group (e.g. after specific sessions; at 3 months follow-up), and reason	- Quantitative descriptive analysis - Field notes	- Interim assessments - End of intervention	- ≥ 70% follow-up completion at 3-month post-randomisation
	- Dose (number of sessions received per participant)	- Field notes	- After each session	- ≥ 70% of participants complete at least 4 of 5 counselling sessions
**Data collection:** To investigate if data collection was possible and conducted accurately	- Completeness (%) for primary and secondary outcomes - Missing values across time points, variables, participants - Data accuracy: Out of range checks; wrong entries	- Quantitative descriptive analysis	- Monitoring during early data collection phase - End of data collection	- ≥ 80% completeness of outcome data
2. **Testing early implementation outcomes and safety of Mommy and Baby Wellness Programme**				
**Feasibility of delivery:** To investigate if lay health care providers can deliver the intervention and session content as intended, and/or if there are any barriers to service delivery	*- (…)* the extent to which a new treatment, or an innovation, can be successfully used or carried out within a given agency or setting [31]	- Qualitative interviews with lay health care providers	- After the 3 months follow-up	- No major barriers to CHW delivery identified in qualitative interviews
	- Intervention fidelity checks (i.e. with regards to session content)	- Fidelity checklist (filled in by intervention supervisors)	- End of sessions	- High intervention delivery scores
**Acceptability of session content**: To assess if Mommy and Baby is suitable to the target population, and if participants and lay health care providers find the intervention and its content acceptable	- *(…)* the perception that a given treatment, service, practice, or innovation is agreeable, palatable, or satisfactory [31]	- Qualitative interviews with participants and lay health care providers	- After the 3 months follow-up	- Participants do not suggest major change to session content; no major barriers to CHW delivery identified in qualitative interviews
**Serious adverse events (SAEs)/adverse events (AEs):** To assess if any safety concerns and risks are linked to the intervention, or the process of delivery	- Number of SAEs/AEs (related and unrelated to intervention)	- Field notes and logs by research assistants	- Continuously during study	- < 10% of participants report adverse events attributable to study procedures or CAP-PRE delivery
3. **Perceived effectiveness of Mommy and Baby Wellness Programme**				
**Perceived effectiveness:** To explore qualitatively how participants perceive the intervention and its impact on drinking and well-being	- Perceived reduction in alcohol misuse and perceived improvement in mental well-being	- Qualitative interviews with participants	- After 3 months follow-up	- Participants suggest that intervention has a positive impact on their drinking and mental health

The progression criteria in Table 1 will be used within a simple go/modify/stop framework to guide decisions about a future definitive trial. If the majority of core feasibility targets (e.g. recruitment, retention, adherence, data completeness and safety) are met or exceeded, and qualitative data indicate good acceptability, we will consider this as ‘go’, proceeding to plan a larger trial with minor refinements. If some key criteria are partially met or borderline (for example, lower than anticipated retention or adherence) but the intervention remains acceptable and safe, we will adopt a ‘modify’ decision, using the feasibility findings to adjust trial procedures or intervention delivery before progression. If several core criteria fall clearly below targets or raise major concerns about feasibility or safety, this will be interpreted as ‘stop’, and plans for a definitive trial will be reconsidered. These decisions will be made by the Project Management Group in consultation with stakeholders, integrating quantitative and qualitative evidence.

## Analyses

### Quantitative data analysis

The statistical analysis will be carried out by a statistician, using SAS 9.4 (SAS Institute Inc., Cary, NC). The reporting of this trial will follow CONSORT guidelines for pilot and feasibility trials [16]. A flow diagram will be produced, depicting the flow of patients through the trial. As this is a Pilot RCT, the main analysis will focus on prespecified feasibility as reported in Table 1. Descriptive statistics will be used to depict the number of potentially eligible participants identified through screening, the recruitment rate (the number and proportion of eligible participants consenting), and reasons for refusal. Uptake of the intervention, retention in the study, and adherence to the intervention will be described. Feasibility of follow-up will be assessed by questionnaire completion rates, and intervention fidelity will be examined using standardised fidelity checklists. Drop-out rates, baseline characteristics, and outcomes at the 3-month end-point will be summarised descriptively. Patterns of dropout and session completion will be used to identify points in the trial or intervention where participation declines, informing refinements to recruitment, scheduling and support procedures. Feasibility indicators will also be described overall and, in an exploratory manner, by key social characteristics (e.g. age, education, socioeconomic status, relationship status). Given the small sample size and feasibility focus, these subgroup summaries will be descriptive rather than powered tests of differential effectiveness but will help identify groups for whom trial procedures or intervention delivery may need adaptation in a future definitive trial. Linear mixed models will be used to explore the direction and potential magnitude of change in clinical outcomes examine whether higher adherence (number of sessions attended) is associated with greater reductions in alcohol use and improvements in mental health outcomes, recognising that these analyses are hypothesis-generating given the small sample size. Where appropriate, linear mixed models with random intercepts for participants and fixed effects for time, trial arm and their interaction (including the amount of missing data) will be used to explore changes in continuous outcomes between baseline and three-month follow-up. These models will be specified parsimoniously (e.g. adjusting only for key baseline covariates if sample size allows) to provide preliminary estimates of effect sizes and variability to inform sample size calculations and outcome selection for a future definitive trial. Together, these analyses will provide evidence to judge whether trial procedures are workable and inform progression to a full RCT.

### Qualitative data analyses

Qualitative data with participants and community health workers will be analysed thematically based on hypotheses of how the intervention influences behaviour, as well as topics and findings that emerge throughout the intervention and analysis. Baseline interviews with women will explore their circumstances and feelings pre-intervention and learn about their attitudes towards drinking while pregnant; their reasons for participating in the program, and their expectations and concerns about both their pregnancies and the programme. Post-intervention interview topics include perceived supportive elements of the programme; potential barriers to uptake and involvement; and how it fits into women’s everyday lives, relationships, and employment. Women’s sense of well-being and agency, knowledge about alcohol-related harms and the perceived usefulness of learned coping strategies will also be investigated. Moreover, other (unintended) effects of the intervention will be explored. The CHWs delivering the counselling will be interviewed about their experiences with the programme; perceptions of women’s involvement and how the programme supports them; and barriers and facilitators for uptake. Qualitative interviews with participants and CHWs will pay particular attention to how stigma, intimate partner violence, social support, transport, childcare, work schedules and other structural constraints shape women’s ability to participate and engage with the intervention, so that potential inequities in access and acceptability can be identified and addressed.

Analysis will be conducted using a structured coding framework developed iteratively by the research team, with regular discussion to ensure reflexivity and consistency. At least two analysts will be involved in coding and theme development. Analysts will first familiarise themselves with the transcripts, then generate initial codes inductively, organise codes into themes, and iteratively review and refine themes in team discussions. NVivo or Word will be used to support data management and coding. Collectively, the analysis will generate nuanced, contextually grounded evidence on the intervention’s acceptability, feasibility of delivery, and perceived effects that may influence the scalability of the intervention.

### Data integration

Qualitative and quantitative findings will be integrated at the interpretation stage. For each feasibility domain (trial feasibility, intervention acceptability and implementation feasibility), quantitative indicators (e.g. recruitment and retention rates, adherence, fidelity scores and data completeness) will be considered alongside themes from participant and provider interviews that speak to barriers, facilitators and perceived impact. This form of triangulation will be used to identify converging evidence as well as discrepancies and will guide recommendations for refining trial procedures and the intervention before a definitive trial.

## Dissemination

Findings from this pilot trial will be disseminated widely to inform future funding applications for a definitive trial, contribute to scientific intervention research on maternal alcohol use and contribute to ongoing policy and service development efforts. Results will be shared through peer-reviewed publications and presentations at local, national and international academic and clinical conferences. Community dissemination events will be held in participating communities to share findings in accessible formats with participants and local stakeholders. In addition, results will be integrated into existing stakeholder meetings with the Western Cape Provincial Department of Health to facilitate dialogue on the potential for future implementation and scale-up.

## Trial status

The study protocol (V.1.0) was approved on 30 April 2024 prior to commencement of formative work. Recruitment to the feasibility study opened on 13 February 2025 with the first participant randomised on the same day. The trial is ongoing and final participants have not completed their last visit prior to submission.

## Discussion

The Mommy and Baby Wellness Programme responds to an urgent need for evidence-based interventions to address alcohol use among pregnant women in South Africa, an often-overlooked and hard-to-reach population due to the social stigma, discrimination, and fear associated with disclosing alcohol use during pregnancy [32]. Despite high rates of alcohol-exposed pregnancies in certain regions of the Western Cape, there are currently no brief, community-delivered, evidence-based counselling interventions tailored to this group. This pilot trial will generate valuable information about the feasibility and acceptability of a CHW-delivered brief psychological intervention designed to reduce alcohol use and improve psychosocial well-being among pregnant women. Findings will be used to inform the design of a future definitive trial evaluating the effectiveness and cost-effectiveness of the intervention, a lay-delivered psychological counselling intervention specifically adapted for the South African context.

This feasibility trial is focused on assessing recruitment, participation, and retention in a vulnerable and stigmatised population, alongside identifying barriers to behaviour change and trial participation. By closely monitoring these feasibility indicators and integrating feedback from process evaluation activities, the trial will help refine strategies for optimising participant engagement and improving implementation fidelity. The process evaluation will also help identify whether further cultural or contextual adaptations are needed to ensure the intervention remains responsive to local beliefs, norms, and service realities. Furthermore, the study will provide critical insight into the suitability of CHWs as delivery agents, and into the preferred locations, timing, and structure of community-based counselling sessions, information that will directly inform future scale-up efforts.

Another important strength of the feasibility trial is its potential policy relevance. The Western Cape Department of Health and Wellness (WCDoHW), which is responsible for health service delivery across the province, has expressed interest in addressing alcohol use among pregnant women, yet lacks the evidence needed to guide intervention rollout. Findings from this study may help shape decisions around who should deliver counselling for alcohol problems (e.g. CHWs), where it should be delivered (e.g. community-based clinics or outreach settings), and how best to structure and support the delivery of such services. Given the sensitivity of alcohol use during pregnancy, the study will also offer insight into how trust and confidentiality can be built between CHWs and participants, and how stigma may or may not shape engagement with community-based interventions. If proven feasible and acceptable, the intervention could be scaled to other provinces and settings within South Africa where similar needs exist. Furthermore, should the pilot demonstrate feasibility, these findings can be leveraged to secure funding for a definitive trial that robustly assesses intervention effectiveness. In the longer term, this work may support policy uptake [33] and integration of community-based alcohol counselling into the broader maternal health system in South Africa.

As with all feasibility studies, there are limitations. The current study is not designed to evaluate intervention effectiveness and no direct comparisons between groups are planned, as the small sample size increases the risk of misleading estimates. Instead, our focus is on testing recruitment and retention procedures, the acceptability and delivery of the intervention, and the feasibility of collecting key outcome measures such as PEth levels and structured self-report data. Additionally, although the intervention has been adapted through an extensive participatory process, its application in other contexts or provinces may require further adaptation to address local service delivery infrastructure and community needs.

Furthermore, while the use of community health workers as interventionists enhances the potential for scalability and sustainability [34], it also introduces variability in fidelity and delivery. We will mitigate this by providing structured training and regular supervision. To ensure fidelity and quality, all counselling sessions will be audio-recorded (with participant consent), and a sample of these recordings will be reviewed by the supervisory team using a structured fidelity checklist. This approach will allow for more rigorous monitoring of adherence to the intervention manual and counselling techniques, and it will inform feedback during supervision sessions. This structured fidelity monitoring strengthens the internal validity of the pilot and will help determine the training and supervision needs of CHWs for a larger trial.

Finally, because participants are recruited from shared community settings, there is potential for contamination between trial arms. To minimise this risk, all Mommy and Baby sessions will be delivered at the research site rather than in participants’ homes and community health workers will be trained not to provide intervention content to control-arm participants.

Ultimately, the current feasibility trial will generate essential data for planning a future full-scale trial. It will offer critical insights into the logistical, contextual, and clinical considerations necessary to deliver a psychological intervention to a socially marginalised group in a resource-constrained health system. If this intervention proves feasible, it has the potential to contribute to long-term improvements in maternal and child health outcomes in South Africa by reducing the harms of prenatal alcohol exposure and strengthening the role of community-based maternal health services.

## Supplementary Information

Additional file 1. SPIRIT 2025 checklist.

## Data Availability

Not applicable.

## References

[1] Bryazka D, Reitsma MB, Griswold MG, Abate KH, Abbafati C, Abbasi-Kangevari M, et al. Population-level risks of alcohol consumption by amount, geography, age, sex, and year: a systematic analysis for the Global Burden of Disease Study 2020. Lancet. 2020;400(10347):185–235. 10.1016/S0140-6736(22)00847-9 PMC9289789 35843246

[2] Shield K, Manthey J, Rylett M, Probst C, Wettlaufer A, Parry CDH, et al. National, regional, and global burdens of disease from 2000 to 2016 attributable to alcohol use: a comparative risk assessment study. Lancet Public Health. 2000;5(1):e51-61. 10.1016/S2468-2667(19)30231-2 31910980

[3] WHO. Alcohol consumption and sustainable development Copenhagen, WHO. 2020. Available from: https://www.euro.who.int/__data/assets/pdf_file/0008/464642/Alcoholconsumption-and-sustainable-development-factsheet-eng.pdf.

[4] Lange S, Probst C, Gmel G, Rehm J, Burd L, Popova S. Global Prevalence of Fetal Alcohol Spectrum Disorder Among Children and Youth: A Systematic Review and Meta-analysis. JAMA Pediatr. 2017;171(10):948–56. 10.1001/jamapediatrics.2017.1919 PMC5710622 28828483

[5] Clarke ME, Gibbard WB. Overview of fetal alcohol spectrum disorders for mental health professionals. Can Child Adolesc Psychiatr Rev. 2003;12(3):57–63. PMC2582751 19030526

[6] Adebiyi BO, Mukumbang FC, Beytell A-M. A guideline for the prevention and management of Fetal Alcohol Spectrum Disorder in South Africa. BMC Health Serv Res. 2019;19(1):809. 10.1186/s12913-019-4677-x PMC6836420 31694624

[7] Jones HE, Browne FA, Myers BJ, Carney T, Ellerson RM, Kline TL, et al. Pregnant and nonpregnant women in Cape Town, South Africa: drug use, sexual behavior, and the need for comprehensive services. Int J Pediatr. 2011. 10.1155/2011/353410. PMC3083847 21541067

[8] Nadkarni A, Weobong B, Weiss HA, McCambridge J, Bhat B, Katti B, et al. Counselling for Alcohol Problems (CAP), a lay counsellor-delivered brief psychological treatment for harmful drinking in men, in primary care in India: a randomised controlled trial. Lancet. 2017;389(10065):186–95. 10.1016/S0140-6736(16)31590-2 PMC5236065 27988144

[9] Samawi L, Petersen Williams P, Myers B, Fuhr DC. Effectiveness of psychological interventions to reduce alcohol consumption among pregnant and postpartum women: a systematic review. Arch Womens Ment Health. 2021. 10.1007/s00737-020-01100-5. 33404702

[10] Nadkarni A, Weiss HA, Weobong B, McDaid D, Singla DR, Park AL, et al. Sustained effectiveness and cost-effectiveness of Counselling for Alcohol Problems, a brief psychological treatment for harmful drinking in men, delivered by lay counsellors in primary care: 12-month follow-up of a randomised controlled trial. PLoS Med. 2017;14(9):e1002386. 10.1371/journal.pmed.1002386 PMC5595289 28898239

[11] Petersen Williams P, Erasmus J, Myers B, Nadkarni A, Fuhr DC. Community-based counselling programme for pregnant women with alcohol problems in Cape Town, South Africa: a qualitative study of the views of pregnant women and healthcare professionals. Front Psychiatry. 2023;14:1203835. 10.3389/fpsyt.2023.1203835 PMC10357510 37484680

[12] Schneider H, Lehmann U. Lay health workers and HIV programmes: implications for health systems. AIDS Care. 2010;22(Suppl 1):60–7. 10.1080/09540120903483042 20680861

[13] Assegaai T, Schneider H, Scott V. Developing a district level supportive supervision framework for community health workers through co-production in South Africa. BMC Health Serv Res. 2021;21(1):337. 10.1186/s12913-021-06350-2 PMC8045385 33853606

[14] Thabane L, Lancaster G. A guide to the reporting of protocols of pilot and feasibility trials. Pilot Feasibility Stud. 2019;5(1):37. 10.1186/s40814-019-0423-8 PMC6393983 30858987

[15] Chan AW, Tetzlaff JM, Altman DG, Laupacis A, Gøtzsche PC, Krleža-Jerić K, et al. SPIRIT 2013 statement: defining standard protocol items for clinical trials. Ann Intern Med. 2013;158(3):200–7. 10.7326/0003-4819-158-3-201302050-00583 PMC5114123 23295957

[16] Eldridge SM, Chan CL, Campbell MJ, Bond CM, Hopewell S, Thabane L, et al. CONSORT 2010 statement: extension to randomised pilot and feasibility trials. BMJ. 2010;355:i5239. 10.1136/bmj.i5239 PMC5076380 27777223

[17] May PA, Gossage JP, Marais AS, Adnams CM, Hoyme HE, Jones KL, et al. The epidemiology of fetal alcohol syndrome and partial FAS in a South African community. Drug Alcohol Depend. 2007;88:259–71. 10.1016/j.drugalcdep.2006.11.007 PMC1865526 17127017

[18] May PA, Blankenship J, Marais AS, Gossage JP, Kalberg WO, Barnard R, et al. Approaching the prevalence of the full spectrum of fetal alcohol spectrum disorders in a South African population-based study. Alcohol Clin Exp Res. 2013;37(5):818–30. 10.1111/acer.12033 PMC3610844 23241076

[19] May PA, Marais AS, de Vries MM, Kalberg WO, Buckley D, Hasken JM, et al. The continuum of fetal alcohol spectrum disorders in a community in South Africa: prevalence and characteristics in a fifth sample. Drug Alcohol Depend. 2016;168:274–86. 10.1016/j.drugalcdep.2016.09.025 PMC5086258 27736681

[20] May PA, De Vries MM, Marais AS, Kalberg WO, Buckley D, Adnams CM, et al. Replication of high fetal alcohol spectrum disorders prevalence rates, child characteristics, and maternal risk factors in a second sample of rural communities in South Africa. Int J Environ Res Public Health. 2017;14(5):522. 10.3390/ijerph14050522 PMC5451973 28498341

[21] Vythilingum B, Roos A, Faure SC, Geerts L, Stein DJ. Risk factors for substance use in pregnant women in South Africa. S Afr Med J. 2012;102(11 Pt 1):851–4. 10.7196/samj.5019 23116742

[22] Petersen Williams P, Jordaan E, Mathews C, Lombard C, Parry CDH. Alcohol and other drug use during pregnancy among women attending midwife obstetric units in the Cape Metropole, South Africa. Adv Prev Med. 2014. 10.1155/2014/871427. PMC3930165 24639899

[23] Sobell LC, Sobell MB. Timeline Follow-Back. Measuring Alcohol Consumption. In: Litten RZ, Allen JP, editors. Totowa: Humana Press; 1992. 10.1007/978-1-4612-0357-5_3.

[24] Varga A, Hansson P, Johnson G, Alling C. Normalization rate and cellular localization of phosphatidylethanol in whole blood from chronic alcoholics. Clin Chim Acta. 2000;299(1–2):141–50. 10.1016/s0009-8981(00)00291-6 10900300

[25] Bush K, Kivlahan DR, McDonell MB, Fihn SD, Bradley KA, Project ftACQI. The AUDIT Alcohol Consumption Questions (AUDIT-C): An Effective Brief Screening Test for Problem Drinking. Arch Internal Med. 1998;158(16):1789–95. 10.1001/archinte.158.16.1789 9738608

[26] Cox JL, Holden JM, Sagovsky R. Detection of postnatal depression. Development of the 10-item Edinburgh postnatal depression scale. Br J Psychiatry. 1987;150:782–6. 10.1192/bjp.150.6.782 3651732

[27] WHO. Measuring health and disability: manual for WHO disability assessment schedule. Geneva, Switzerland: World Health Organisation; 2010.

[28] Berman AH, Bergman H, Palmstierna T, Schlyter F. Drug Use Disorders Identification Test (DUDIT): manual. Karolinska Institutet; 2005. 10.1159/000081413 15608468

[29] van der Boor CF, Taban D, Tol WA, Akellot J, Neuman M, Weiss HA, Greco G, Vassall A, May C, Nadkarni A, Kinyanda E, Roberts B, Fuhr DC. Effectiveness and cost-effectiveness of a transdiagnosticintervention for alcohol misuse and psychological distress in humanitarian settings: study protocol for a randomisedcontrolled trial in Uganda. Trials. 2024;25(1):148. 10.1186/s13063-024-07980-7 PMC10900822 38414078

[30] Mellor K, Albury C, Dutton SJ, Eldridge S, Hopewell S. Recommendations for progression criteria during external randomised pilot trial design, conduct, analysis and reporting. Pilot Feasibility Stud. 2023;9(1):59. 10.1186/s40814-023-01291-5 PMC10105402 37061720

[31] Proctor E, Silmere H, Raghavan R, Hovmand P, Aarons G, Bunger A, et al. Outcomes for implementation research: conceptual distinctions, measurement challenges, and research agenda. Adm Policy Ment Health. 2011;38(2):65–76. 10.1007/s10488-010-0319-7 PMC3068522 20957426

[32] Stone R. Pregnant women and substance use: fear, stigma, and barriers to care. Health & Justice. 2015;3(1):2.

[33] Adebiyi BO, Mukumbang FC, Beytell AM. Policy requirements for the prevention and management of fetal alcohol spectrum disorder in South Africa: a policy brief. Front Public Health. 2021;9:592726. 10.3389/fpubh.2021.592726 PMC8082060 33937161

[34] Pallas SW, Minhas D, Pérez-Escamilla R, Taylor L, Curry L, Bradley EH. Community health workers in low- and middle-income countries: what do we know about scaling up and sustainability? Am J Public Health. 2013;103(7):e74-82. 10.2105/AJPH.2012.301102 PMC3682607 23678926

